# What leads to a Public Health Emergency of International Concern? A comparison of the 2026 Ebola and hantavirus outbreaks

**DOI:** 10.1093/jtm/taag053

**Published:** 2026-06-23

**Authors:** Adam T Craig, Meru Sheel, Eric J Nilles, Colleen L Lau

**Affiliations:** Operational Research and Decision Support for Infectious Diseases (ODeSI) Program, Frazer Institute, The University of Queensland, Brisbane, QLD, Australia; School of Public Health, Faculty of Medicine and Health, The University of Sydney, Sydney, NSW, Australia; Sydney Infectious Diseases Institute, Faculty of Medicine and Health, The University of Sydney, Sydney, NSW, Australia; Brigham and Women’s Hospital, Harvard Medical School, Boston, MA, USA; Operational Research and Decision Support for Infectious Diseases (ODeSI) Program, Frazer Institute, The University of Queensland, Brisbane, QLD, Australia

## Abstract

Why has WHO declared the 2026 Ebola outbreak in the Congo a global emergency, but not the deadly hantavirus cruise ship outbreak? This article explains how transmission dynamics, epidemic potential, impact on travel and trade and geopolitics shape the decision to declare a ‘Public Health Emergency of International Concern.’

The simultaneous emergence in May 2026 of two severe outbreaks, affecting multiple countries, caused by pathogens with similar early case fatality rates but different transmission characteristics, presents an opportunity to examine how the World Health Organization (WHO) operationalises the declaration of a Public Health Emergency of International Concern (PHEIC).

On 16 May 2026, the World Health Organisation announced that Ebola disease caused by Bundibugyo virus in the Democratic Republic of the Congo (DRC) and Uganda met the threshold for a PHEIC. At the time, WHO reported eight laboratory-confirmed cases, 246 suspected cases and 80 suspected deaths (case-fatality rate: 33%) affecting health zones in Ituri Province, DRC, with confirmed exported cases in Kampala, Uganda.^1^ Just days earlier, a cluster of severe respiratory illness caused by the Andes strain of hantavirus among passengers of the cruise ship *MV Hondius* had also gained international attention and by 13 May 2026, 11 cases and three deaths (case-fatality rate: 27%) among passengers and crew had been reported, with evidence suggesting human-to-human transmission on board^2^ (Figure 1). Yet despite its severity, multinational implications and high early case fatality rate, the hantavirus event did not result in WHO declaration of a PHEIC.

**Figure 1:**
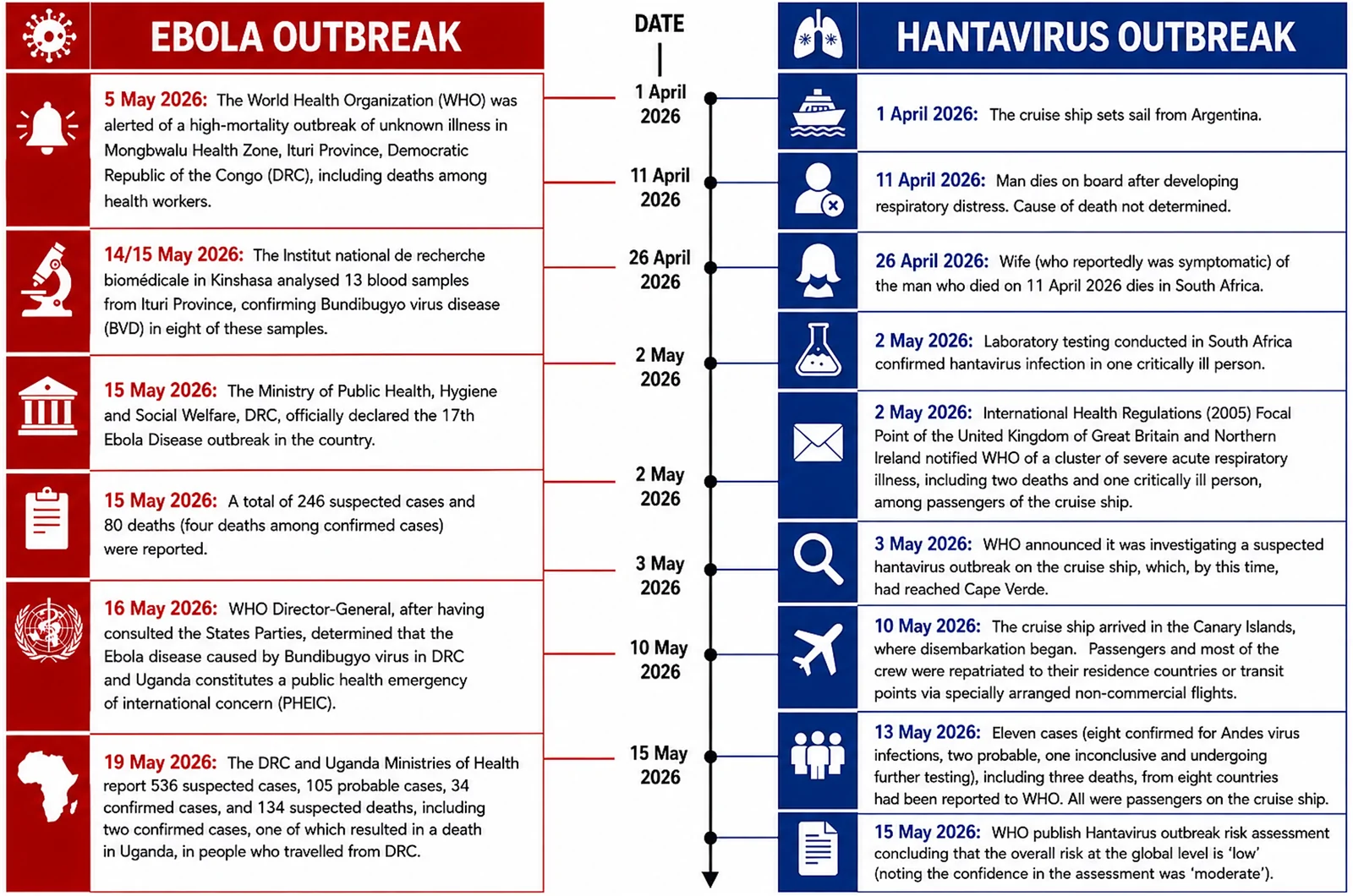
Timeline of major developments in the 2026 BVD and Andes hantavirus outbreaks, 1 April–19 May 2026.

This contrast raises an important public health question: why does one important international public health event trigger a PHEIC while another does not? The answer lies not simply in case fatality rates or disease severity, but in differences in transmission dynamics, epidemic potential, uncertainty, likelihood of international spread and the need for a coordinated international response.

## Why the 2026 Bundibugyo virus disease (BVD) outbreak triggered WHO’s declaration of a PHEIC

The 2026 BVD outbreak possessed several characteristics that collectively justified its designation as a PHEIC. Ebola is readily transmitted through close contact with infected bodily fluids, contaminated materials and the bodies of deceased patients; and, in settings where infection prevention and control systems are weak, transmission can rapidly intensify within households and healthcare facilities.^2^ By 15 May 2026, the DRC BVD outbreak had already spread across international borders into Uganda,^1^ meeting one of the key criteria for declaring a PHEIC under the International Health Regulations (2005).^3^

Unlike many zoonotic diseases that remain geographically constrained, Ebola outbreaks can escalate into regional epidemics when containment is delayed. The memory of the 2014–6 West African Ebola epidemic,^4^ which infected 28 000 people and caused over 11 000 deaths, continues to shape global risk perception and assessment.^4^^,^^5^

## The Bundibugyo strain lacks medical countermeasures

A further concern is that the 2026 Ebola outbreak involves the Bundibugyo virus, a rare species of Ebola virus. Existing Ebola vaccines and therapeutics were primarily developed for the Zaire strain and have uncertain effectiveness against BVD.^6^ This limits the public health tools available to respond to and contain the current outbreak.

During recent outbreaks caused by the Zaire strain, ring vaccination strategies and antibody treatments played central roles in containment. In contrast, the BVD outbreak requires authorities to rely on traditional public health measures, including isolation, border surveillance, contact tracing, community engagement and infection prevention and control, all of which are complex to implement and sustain without breaches. Clinical management will rely predominantly on supportive care. This absence of countermeasures increases uncertainty about the outbreak’s trajectory and the likelihood of containment.

## The BVD outbreak is occurring in a fragile and conflict-affected area

The outbreak emerged in eastern DRC, an area affected by conflict, population displacement, weak infrastructure and limited healthcare resources.^7^ Conflict settings create ideal conditions for uncontrolled transmission: surveillance systems are weakened, population movement is difficult to track, healthcare workers face security risks and communities may distrust authorities.^7^

The WHO explicitly noted concerns that the outbreak may have been spreading undetected for weeks before formal recognition,^1^ which increases the probability of already widespread transmission.

## Urban transmission and cross-border risk of BVD

The detection of cases in Kampala, Uganda,^1^ a city of 4.8 million, when including the wider metropolitan boundary and concerns for further spread into other major centres, significantly escalated international anxiety.^1^ Urban Ebola transmission is particularly dangerous because population density accelerates contact and transmission rates. Further, urban centres act as ‘hubs’ for domestic and international travel, increasing the risk that the disease will spread, including across borders.^8^

Reports of infections among healthcare workers further intensified concerns.^1^ Healthcare worker infections are epidemiologically important because they indicate gaps in infection prevention and, potentially, the collapse of already fragile health services. Fears of hospital-associated transmission were a factor that undermined community willingness to seek care during past Ebola outbreaks.^9^

## Why the hantavirus Andes strain outbreak did not trigger a PHEIC

In contrast, the Hantavirus outbreak, despite having a similarly high case-fatality rate, has not been declared a PHEIC. Why?

A key distinction lies in transmission dynamics. Hantavirus outbreaks are typically associated with identifiable ecological conditions, with human infection occurring through zoonotic spillover from rodents.^10^ In most settings, humans are considered incidental or dead-end hosts, resulting in short and geographically contained transmission chains that limit broader epidemic potential.^2^ The 2026 hantavirus Andes strain outbreak differs in one important respect, with preliminary evidence suggesting that human-to-human transmission has occurred onboard the ship. Early genetic sequencing supports this possibility, demonstrating near-identical viral sequences across multiple cases.^10^ Nevertheless, unlike Ebola, hantavirus infections are generally not easily transmitted between humans and, historically, have not demonstrated the capacity for sustained, rapid, or widespread epidemic growth.^4^

Another distinguishing factor is that, because human-to-human transmission is rare, hantavirus outbreaks rarely overwhelm health systems. Severe disease, including Hantavirus Pulmonary Syndrome, may require intensive care; however, outbreaks have not typically produced the degree of health system disruption seen with Ebola, including the need for specialized isolation facilities, extensive contact tracing, complex burial management and protection of healthcare workers operating in high-risk environments.^7^ Even relatively small Ebola outbreaks can rapidly overwhelm fragile health systems as demand outstrips the capacity to deliver care.^7^

## Ebola carries greater international political and social consequences

The declaration of a PHEIC is not purely biomedical; it also reflects concerns about economic upheaval, political risk and disruption of travel and trade. The strength of PHEIC declarations lies in their ability to rapidly mobilize international coordination and funding and to accelerate the development of therapeutics and diagnostics under emergency authorisation.^11^

Ebola has become symbolically associated with high-consequence epidemic risk. Images of haemorrhagic disease, overwhelmed treatment centres and healthcare workers in full protective equipment carry substantial emotional and political weight. The 2014–6 West African epidemic fundamentally reshaped international perceptions of Ebola, exposing major weaknesses in global preparedness and prompting significant reforms to the WHO’s emergency response systems. The experience during these outbreaks created strong institutional incentives for early and precautionary international action when Ebola outbreaks emerge.^12^ By contrast, hantavirus, because it is not efficiently transmitted between humans or at scale, lacks the same potential for widespread international disruption and transnational destabilization.

## Conclusion

The contrast between the 2026 BVD and hantavirus Andes strain outbreaks illustrates a fundamental principle of global health security: public health emergencies are determined not solely by disease severity, but by epidemic potential and socio-political factors. A disease becomes a global emergency when it combines high mortality with efficient human-to-human transmission, particularly where cases are not rapidly detected and isolated. The Ebola outbreak meets these criteria. By contrast, although the hantavirus outbreak has tested International Health Regulations mechanisms and provided a contemporary example of global coordination and risk assessment under the International Health Regulations framework, it has not demonstrated the same epidemic characteristics.

## Data Availability

The data underlying this article are available in the article.
